# A Combinative Assembly Strategy Inspired Reversibly Borate-Bridged Polymeric Micelles for Lesion-Specific Rapid Release of Anti-Coccidial Drugs

**DOI:** 10.1007/s40820-020-00495-1

**Published:** 2020-07-25

**Authors:** Hao Cheng, Huaqing Zhang, Gujun Xu, Jin Peng, Zhen Wang, Bo Sun, Djamila Aouameur, Zhechen Fan, Wenxin Jiang, Jianping Zhou, Yang Ding

**Affiliations:** 1 State Key Laboratory of Natural Medicines, Department of Pharmaceutics, China Pharmaceutical University, 24 Tongjiaxiang, Nanjing, 210009 People’s Republic of China; 2 Department of Radiation Oncology, Lineberger Comprehensive Cancer Center, Carolina Center for Cancer Nanotechnology Excellence, Carolina Institute of Nanomedicine, University of North Carolina at Chapel Hill, Chapel Hill, NC 27599 USA

**Keywords:** Combinative assembly strategy, Borate-bridged micelles, Dual-stimuli-triggered release, Lesion-specific location, Coccidiosis control

## Abstract

**Highlights:**

- A combined assembly strategy from hydrophobicity-driving and reversible borate bridges is proposed for high drug-loading efficiency and superior stability.
- Intestinal environment-triggered drug delivery system represents an effective treatment for local infection due to the site-specific targeting and shuttling of drugs.
- The reduced dosage brought by the drug-loading micelles could solve the problem of drug residue in breeding industry.

**Abstract:**

Stimuli-triggered drug delivery systems hold vast promise in local infection treatment for the site-specific targeting and shuttling of drugs. Herein, chitosan conjugates (SPCS) installed with sialic acid (SA) and phenylboronic acid (PBA) were synthesized, of which SA served as targeting ligand for coccidium and reversible-binding bridge for PBA. The enhanced drug-loading capacity of SPCS micelles was attributed to a combination assembly from hydrophobicity-driving and reversible borate bridges. The drug-loaded SPCS micelles shared superior biostability in upper gastrointestinal tract. After reaching the lesions, the borate bridges were snipped by carbohydrates under a higher pH followed by accelerated drug release, while SA exposure on micellar surface facilitated drug cellular internalization to eliminate parasites inside. The drug-micelles revealed an enhanced anti-coccidial capacity with a higher index of 185.72 compared with commercial preparation. The dual-responsive combination of physicochemical assembly could provide an efficient strategy for the exploitation of stable, safe and flexible anti-infectious drug delivery systems.
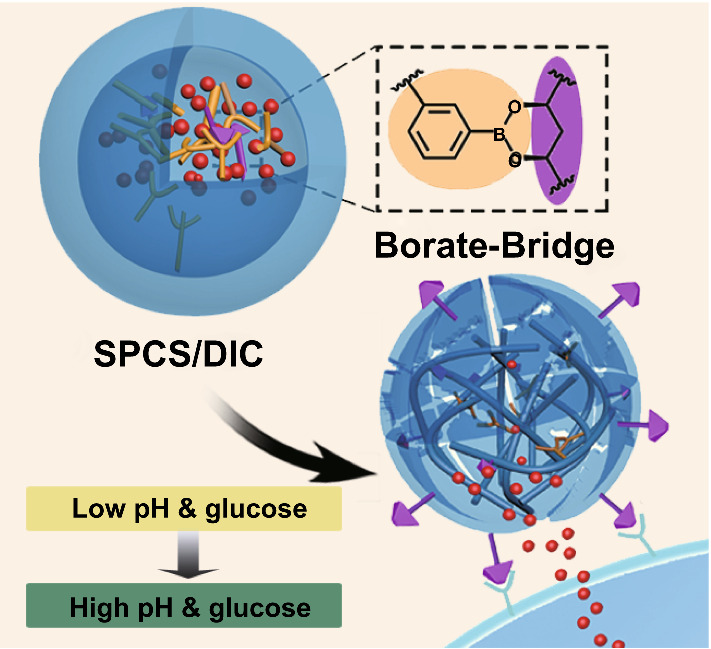

**Electronic supplementary material:**

The online version of this article (10.1007/s40820-020-00495-1) contains supplementary material, which is available to authorized users.

## Introduction

Livestock infectious diseases caused by various microorganisms are widespread and economically significant, the outbreak of which may induce serious food safety and animal food storage issues [1–3]. Thus, the prevention and control of infectious diseases have raised worldwide concern, especially in large-scale poultry farming, which is much more vulnerable to such diseases like coccidiosis due to intensive feeding [4]. The coccidiosis is of high sensitivity to chemotherapy during the schizozoite period of oral–fecal life cycle [5]. Aiming at this key period, numerous active ingredients are introduced for coccidiostats, among which triazine benzacetonitril anti-coccidial drugs are of broad spectrum and high efficacy [6]. As for practical applications, the commercial coccidiostats are widely applied for anti-coccidiosis prophylaxis and treatment in the form of solution, suspension and premix forms [7].

Obviously, the above commercial preparations of coccidiostats center on drug solubilization and absorption enhancement, while losing the sight of pathogenic locations and corresponding lesion-specific drug delivery. *Eimeria tenella* (*E. tenella*), one of the most common and harmful coccidia, mainly infected cecum section of poultry and caused severe damage to intestinal epithelium [8]. The microenvironments of gastrointestinal tract differentiated from intestinal segments in various aspects, including intestinal contents, and different degradants generated by digestion and pH values. Consequently, the environmental variations could be introduced as triggers for rapid drug release at infected lesions. Recently, phenylboronic acid (PBA) has been extensively introduced for flexible regulation of various microenvironment factors [9], such as reactive oxygen species (ROS) [10], sugars [11, 12], adenosine triphosphates [13] and pH variations [14, 15], due to the reversible binding with diverse biomolecules. Sugars like glucose that is rich in intestinal tract can favor borate complexion with hydroxyl groups [10, 16]. The stability of borates is dramatically dependent on pH values and the concentration of cis-diols. Along with pH decrease, most of the borates become unstable and sensitive to hydrolysis. According to Kazunori Kataoka’s researches, the binding affinity profile of phenylboronic acid (PBA) with sialic acid (SA) is unique and intense, which is less affected by pH variations and gives higher affinity under acidic conditions [17, 18].

Chitosan (CS) and its derivations from natural polysaccharide are widely regarded as a safe drug excipient with diverse biological functions including intestinal absorption enhancement, antibiosis, anti-inflammation, anti-oxidation, bioadhesion and immune-stimulation [19–21]. In poultry production, CS was commonly applied as feed supplement which was beneficial to infection prevention, laying intensity and egg quality promotion [22]. However, several drawbacks of CS that could not be ignored were insufficient drug-carrying capacity and low solubility at neutral pH. Due to the presence of reactive amine and hydroxyl groups on CS skeleton, various derivatives can be designed and synthesized for efficient drug shielding and shuttling [23].

**Scheme 1:**
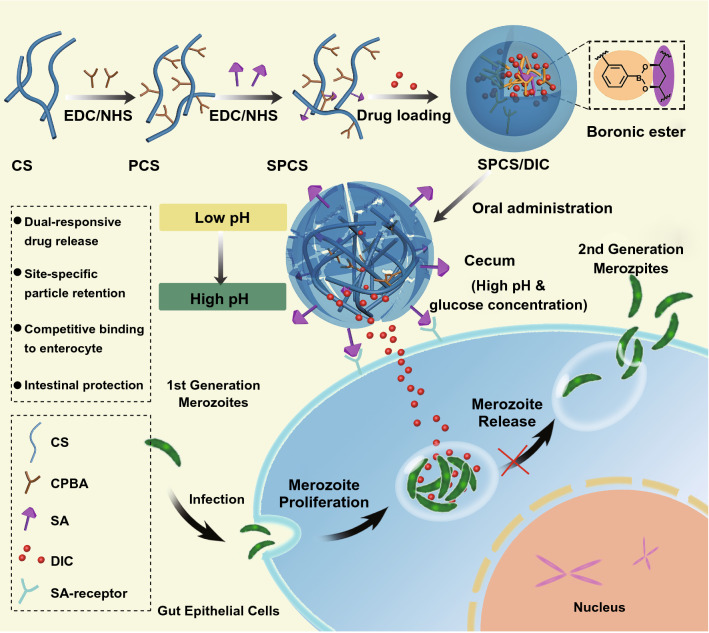
Schematic illustration of SPCS copolymers and its DIC-loaded micelles for peroral lesion-specific coccidiostats delivery. The SPCS copolymer was synthesized by CPBA and SA installation on CS skeleton, and the DIC-loaded SPCS micelles were prepared by combinational self-assembly. After oral administration to coccidian-infected chicken, SPCS/DIC micelles maintained stable until arriving at colon site. The payloads were rapidly released from the micelles triggered by elevatory pH value and glucose concentration. Meanwhile, the SA moieties were transferred on to micelles surface for enhanced lesion-specific accumulation

In this study, we designed a novel chitosan-based nanovehicle for dual-stimuli-triggered site-specific delivery of anti-coccidial agents. As illustrated in Scheme 1, the amphipathic SA-PBA-CS (SPCS) copolymer was fabricated by sequential installation of PBA and SA moieties onto CS skeleton, which was applied to bearing more diclazuril (DIC) due to the intense hydrophobic interaction. SA molecules were introduced for reversible borate bridges in order to provide a superior stability of SPCS micelles accompanied with adjustable drug release profile. The DIC-loaded micelles were expected to remain stable before reaching the infected cecum site, while the in-core borate bridges were snipped by high-concentrated glucose under high pH value of cecum, generating a loose nanostructure and accelerated payload release. Moreover, the detached SA molecules would be transferred onto the surface due to the hydrophilic properties and bind with E-selectin overexpressed on infected cells, which can facilitate cellular conglutination and internalization for eliminating pathogen inside cells. Of note, the nature bacteriostatic activity of chitosan can also prevent occurrence of secondary infection and promote wound healing. This dual-stimuli responsive micellar system offers an efficient nanocarrier platform with great potential for site-specific delivery of anti-infectious agents.

## Experimental Section

### Materials and Reagents

Chitosan (molecular weight = 50 kDa and 90% deacetylated) was purchased from Golden-Shell Pharmaceutical Co., Ltd. (Zhejiang, China). 1-(3-Dimethylaminopropyl)-3-ethylcarbodiimide hydrochloride (EDC·HCl) and N-Hydroxysuccinimide (NHS) were purchased from Aladdin Reagent Inc. (Shanghai, China). 3-Carboxyphenylboronic acid (CPBA) was obtained from Ark Pharm Inc. (Chicago, USA). *N*-Acetylneuraminic acid was purchased from Aladdin Reagent Inc. (Shanghai, China). Diclazuril was purchased from Zhejiang Guobang Pharmaceutical Co., Ltd (Zhejiang, China), and commercially available suspensions of diclazuril were purchased from Jiangxi Bolai Pharmacy Co., Ltd (Jiangxi, China). All chemicals and reagents were of analytical or HPLC grade without further purification unless stated.

### Synthesis and Characterization of SPCS Conjugate

The PCS polymer was synthesized through an amide reaction as previously reported with slight modifications. In brief, CPBA in methanol was blended with EDC and NHS, and the reaction mixture was stirred for 30 min in ice bath. Then, the mixture was added into acidic CS solution) dropwise followed by continuous stirring at room temperature for 24 h. The reaction products were dialyzed against distilled water and lyophilized to obtain PCS polymers. The chemical constructions of the products were determined by ^1^H NMR spectra, XPS spectra and Fourier-transform infrared spectroscopy (FTIR) spectra. The molar ratio of CPBA molecules to reactive chitosan monomer was adjusted as 0.5, 1.0, 1.5 and 2.0, and the corresponding products were named as P_0.5_CS, P_1.0_CS, P_1.5_CS and P_2.0_CS. The grafting degrees of CPBA were determined by ninhydrin colorimetry.

PCS polymers were modified with sialic acid (SA) moiety to obtain SPCS conjugates. Briefly, SA was dissolved in methanol. EDC and NHS were added to the solution and stirred in ice bath for 30 min. Next, PCS polymers were dissolved in water and the activated SA solution was added by drop and stirred at room temperature for 24 h. The obtained SPCS conjugates were purified by dialysis, and chemical structures of SPCS were detected by ^1^H NMR spectra, XPS spectra and FTIR spectra. P_1.0_CS and P_1.5_CS were used to synthesize the SPCS copolymers at 0.1, 0.2, 0.5 and 1.0 ratios of CPBA to SA. The generated products were named as S_0.1_P_1.0_CS, S_0.2_P_1.0_CS, S_0.5_P_1.0_CS, S_1.0_P_1.0_CS, S_0.1_P_1.5_CS, S_0.2_P_1.5_CS, S_0.5_P_1.5_CS and S_1.0_P_1.5_CS. The grafting degrees of SA moieties were determined by ninhydrin colorimetry.

The CMC of SPCS conjugate in distilled water was evaluated through fluorescence spectrophotometry using pyrene as a probe. Briefly, pyrene solution (100 μL, 60 μM) in acetone was added to a series of 10-mL volumetric flasks, and then, acetone was completely evaporated in vacuum. SPCS conjugate aqueous solution (1000 μg mL^−1^) of serial volumes was transferred into the flasks and diluted with water to 10 mL to get SPCS concentrations ranging from 0.001 to 1000 μg mL^−1^. Samples were sonicated in water bath for 30 min first, then incubated in 50 °C for 1 h and finally left to equilibrate for 12 h at room temperature. Pyrene fluorescence spectra of each sample were determined by using a fluorescence spectrophotometer (RF-5301 PC; Shimadzu Corp, Kyoto, Japan) at an emission wavelength of 390 nm, and the slit-widths of both excitation and emission were 3 nm. The CMC was estimated as the intersection point when extrapolating the intensity ratio *I*_338_/*I*_333_ at low and high concentration regions.

Moreover, blank SPCS micelles were prepared and incubated in various media with different pH and glucose concentrations. Briefly, SPCS polymers were dissolved in 3 mL of distilled water, followed by sonication for 20 min with a probe-type ultrasonicator (JY 92-2D, Ningbo Scientz Biotechnology Co., Ltd., Jiangsu, China) at 100 W. The generated suspensions were filtered with microfiltration to collect the blank SPCS micelles. SPCS micelles were dispersed in 20 mM of glucose and free media with different pH values of 2.0, 5.5 and 7.0. The diameter of each sample was determined after incubation for 2 h.

### Characterization of DIC-Loaded Micelles

The particle size, polydispersity index (PDI) and zeta potential (ZP) of DIC-loaded nanoparticles were determined by a dynamic light scattering analyzer (DLS, BI-200SM, Brookhaven Instruments Crop., USA). The morphology of SPCS micelles was visualized under transmission electron microscopy (TEM, H-7000, Hitachi, Japan) operated upon 100 kV of the accelerating voltage. In order to investigate the storage stability of SPCS/DIC micelles, the samples were dispersed in PBS solution and stored at 4 °C. Changes in particle size and PDI were monitored for a week.

Drug loading (DL) and entrapment efficiency (EE) were determined by high-performance liquid chromatography (HPLC, Shimadzu LC-2010, Kyoto, Japan). DIC-entrapped SPCS micelles (60 μL) were diluted with tetrahydrofuran (THF) to 3 mL and vortexed for 10 min to disassemble the micelles and liberate DIC. The samples were then centrifuged at 12,000 rpm for 30 min. The supernatant was collected and filtered through 0.22 μm organic filter. Filtered solution (20 μL) was subjected to HPLC analysis (Lichrospher™ C18 column, 5 µm particle size, 250 × 4.6 mm^2^) for DIC concentration at 277 nm wavelength, with 57% (v/v) acetonitrile aqueous solution containing 0.1% phosphoric acid as mobile phase at a flow velocity of 1.0 mL min^−1^. DL % and EE % were calculated according to Eqs. (1) and (2), respectively.

1 $${\text{DL(\%)}} = \frac{{{\text{Mass}}\;{\text{of}}\;{\text{DIC}}\;{\text{in}}\;{\text{micelles}}}}{{{\text{Total}}\;{\text{mass}}\;{\text{of}}\;{\text{micelles}}}} \times 100{\text{\% }}$$

2 $$E ( {\text{\%)}} = \frac{{{\text{Mass}}\;{\text{of}}\;{\text{DIC}}\;{\text{in}}\;{\text{micelles}}}}{{{\text{Mass}}\;{\text{of}}\;{\text{DIC}}\;{\text{fed}}\;{\text{initially}}}} \times 100{\text{\% }}$$

### pH-Sensitive Binding of PCS to Glucose and SA

The binding of sugar to PBA has impact on the fluorescence of PBA with a concentration dependent manner. Thus, the binding constants between PCS and various sugars like glucose (Glu) and SA can be evaluated. Fluorescence spectra of PCS dissolved in phosphate buffer solution (0.1 M) containing different concentrations of sugars (from 0 to 50 mm) were recorded at pH 5.5 and 7, with an excitation at 371 nm and a slit width set to 5 nm. Emission scans were taken from 380 to 550 nm, and the maximum emission intensities were peaked at 430 nm. The data were fit by the Stern–Volmer equation. The pKa value of SPCS was measured by potentiometric titration. Briefly, 20 mg of SPCS was dissolved in water and the generated solution was adjusted to about pH 2.0 followed by titration with 0.01 M standard NaOH solution. The pH values were recorded, and the pKa value was calculated by differential method.

### X-Ray Diffraction and Differential Scanning Calorimetric Analysis

To determine the existential state of the entrapped DIC and DIC-conjugate relationship and interaction, X-ray diffraction spectroscopy (XRD) and differential scanning calorimetric (DSC) were applied on DIC, physical mixture of DIC-conjugate and DIC/conjugate micelles, respectively. XRD analysis was accomplished by a powder diffraction meter (XD-3A, Bruker, Germany) with Cu K-alpha radiation. Samples were scanned from 5° to 40° at scanning speed of 1° min^−1^ and a step size of 0.05°. DSC analysis was carried out with a differential scanning calorimeter (DSC 204, NETZSCH, Germany) and the samples were heated at a rate of 10 °C min^−1^ and the thermograms were recorded from 40 to 300 °C under a constant protective nitrogen atmosphere.

### In Vitro pH and Glucose-Triggered DIC Release

In vitro DIC release behaviors from SPCS/DIC micelles were studied in PBS solutions containing 0.5% (v/v) of Tween 80 using a dialysis method. In brief, 1 mL of each sample containing 0.2 mg mL^−1^ of DIC was transferred into a dialysis bag (MWCO 12,000–14,000) and submersed in 200 mL of dissolution media under shaking at 100 rpm and 37 °C. Dissolution media with different pH value of 2.0, 5.5 and 7.0 were used to simulate pH environment of chicken gizzard, small intestine and cecum, respectively. To investigate glucose-responsive release of DIC, glucose concentration of 20 and 50 mM was applied to stimulate preprandial and postprandial glucose concentrations in gastrointestinal tract. At predesigned time intervals, 2 mL of release medium was sampled and an equal volume of fresh corresponding medium was added. DIC contents were detected by HPLC. The free DIC dissolved in 30% DMF (0.2 mg mL^−1^) was used as control. To investigate the impact of surfactant on DIC release behavior, SPCS/DIC micelles were dispersed in 0.5% Tween 80 solution and preincubated for 6 h, and then, the SPCS/DIC samples were used to study the release profiles. We further studied the stability of the micelles against surfactant to verify the structural changes. Briefly, PCS/DIC and SPCS/DIC micelles were dispersed in the media containing 0.5% and 2% Tween 80, respectively. The particle size and PDI of each sample were monitored for 24 h.

### Cell Culture

For cell studies, human colon cancer cell line Caco_2_ cells (Shanghai Institute of Biochemistry and Cell Biology, Chinese Academy of Sciences, Shanghai, China) were cultured in DMEM medium supplemented with 10% FBS, 100 U mL^−1^ penicillin, and 100 μg mL^−1^ streptomycin at 37 °C under an atmosphere of 5% CO_2_ and 95% relative humidity.

### In Vitro Cellular Uptake of SPCS Micelles

The cell uptake of SPCS micelles was visualized by inverted fluorescence microscope (Ts2-FL, Nikon, Tokyo, Japan) and flow cytometry (FCM, BD FACS Calibur, USA). The fluorescence marker coumarin-6 (C6) was encapsulated into SPCS micelles (SPCS/C6) and PCS micelles (PCS/C6) to replace the DIC for cell tracking. Human colon cancer Caco_2_ cells were cultured in 24-well plates at a density of 1 × 10^5^ cells per well and further cultured for 24 h. The cells were incubated with SPCS/C6 micelles and PCS/C6 micelles (50 ng mL^−1^, C6) at 37 °C for 1, 3 and 6 h, respectively. Moreover, cells incubated with SPCS/C6 micelles in glycoprival DMEM cell culture medium were used as control to investigate glucose-responsive SA eversion. At the predesigned time point, the medium containing micelles was removed, and the cells were washed and fixed with paraformaldehyde solution (4%, v/v) for 15 min, followed by nuclear staining with DAPI. Finally, all the cell samples were immediately observed using inverted fluorescence microscope.

For FCM analysis, Caco_2_ cells were seeded into 24-well plates at a density of 1 × 10^5^ cells per well. After 24 h of culture, culture medium containing SPCS/C6 and PCS/C6 micelles was used to culture the cells with the sugar-free medium containing SPCS/C6 as control. At the same time intervals, the culture medium was removed and the cells were washed with cold PBS for three times. At last, all cell groups were resuspended in 200 μL of PBS and analyzed using flow cytometry.

### In Vitro Cytotoxicity Assay

In vitro cytotoxicity of free DIC, blank micelles and DIC-encapsulated micelles were evaluated by 3-(4,5-dimethylthiazol-2-yl)-2,5-diphenyltetrazolium bromide (MTT) assay. Caco_2_ cells were planted in 96-well plate at a density of 5 × 10^3^ cells per well and cultured for another 24 h. The cells were treated with free DIC, PCS/DIC micelles, PCS + DIC mixture, SPCS/DIC micelles and SPCS + DIC mixture at a range of DIC concentrations (0.001–100 μg mL^−1^), or PCS and SPCS blank micelles at equivalent concentrations of corresponding drug-loaded micelles for 48 h. Thereafter, 20 µL of MTT solution (5 mg mL^−1^) was added to each well and incubated with cells for another 4 h at 37 °C. The culture medium was then removed, and the formed dark blue formazan crystals were dissolved with 100 µL of DMSO. The absorbance at 570 nm was recorded using a microplate reader (EL800, BIO-TEK Instruments Inc., USA). Untreated cells were used as control, and cell viability (%) was calculated according to Eq. (3).

3 $${\text{Cell}}\;{\text{viability}}(\% ) = \frac{{A_{\text{test}} }}{{A_{\text{control}} }} \times 100\%$$

### Pharmacokinetic Assessment of SPCS/DIC

Experimental animals were raised in laboratory animal room. Chickens were provided with enough water and fed feeds without any anti-coccidial drugs or growth additives. Chickens were raise for 1 week before the trials to be adapted to the new environment. Fifteen chickens were randomly selected from twenty healthy male Wen’s broiler chickens and divided into 3 groups of 5 per group. They were set up as DIC/SPCS group, DIC/PCS group and DIC suspension group. The remaining blank group was used to collect blank blood. Chickens were fasted for 12 h before administration, but with free drinking water. In this experiment, a single dose was administered orally and animals were divided into three groups of three chicken per group. These groups were treated with DIC/SPCS solution, DIC/PCS and commercially available DIC/CMC-Na suspensions, respectively, at the single oral dose of 15 mg/(kg·bw) drug (calculated by DIC), which were directly poured into the crop.

Blank blood samples were taken from the wing vein of chickens in blank group, 10 mL per feather. The collected blood was placed in a heparin-containing centrifuge tube and carefully mixed. The blank samples collected in this experiment were used to plot the standard curve of DIC in blood. Experimental blood samples were taken from the wing vein of chickens in experimental group at certain time points (0, 5, 15, 30, 45 min and 1, 2, 3, 4, 8, 12, 24, 36, 48, 72 h) post-oral administration. All the samples were centrifuged at 4000 r min^−1^ for 10 min, and the supernatant plasma was collected and then stored at − 20 °C for the further use.

According to previous reports, HPLC was used as a diclazuril content detection method using an ODS C18 column as a detection column. HPLC mobile phase was acetonitrile/0.1% trifluoroacetic acid/water = 57/20/23 (v/v/v), with flow rate of 1 mL min^−1^, column temperature of 30 °C, detection wavelength at 280 nm. Accurately pipette 0.1 mL of drug-containing plasma sample into a microcentrifuge tube, in which 0.1 mL diazepam solution (50 μg mL^−1^) was added beforehand. Vortex it and add 0.4 mL MTBE, mixed for 5 min at high speed, and then centrifuged at 12,000 r min^−1^ for 10 min in a high-speed centrifuge. The organic phase was accurately pipetted for further experiments.

Plasma concentration–time curves were processed by using drug statistical software (DAS, Chinese Mathematical Drug Quantity Professional Committee), and pharmacokinetic parameters were calculated by simulating the plasma drug concentration using non-compartmental modeling approaches.

### In Vivo Biodistribution and anti-coccidial Efficacy

All animal experiments were conducted under a protocol approved by China Pharmaceutical University Ethics Committee and in accordance with the National Institutes of Health guide for the care and use of Laboratory animals (NIH Publications No. 8523, revised 1985). One-day-old Wen’s male broilers were purchased from the local hatchery (Nanjing, China) and adapted for 14 days in a coccidia-free environment. The infective coccidial oocysts were isolated and purified from the cecal contents of chickens infected with *E. tenella*. The purified coccidia oocysts were diluted with distilled water to a concentration of 1.0 × 10^5^ spores mL^−1^ and inoculated chicken with 1 mL of coccidiosis oocyst solution.

After infection for 48 h, the chickens were orally administrated with PCS/DiR and SPCS/DiR micelles at a DiR dosage of 0.5 mg kg^−1^. The animals were killed at 4-h post-administration, and the intestinal tracts were excised for ex vivo imaging. Afterward, 60 infected chickens with similar body weights were randomly divided into 6 groups: infected group, DIC/PCS micelles (coccidiosis infection and treated with micellar preparation), DIC suspension group (coccidiosis infection and treated with commercial DIC suspension), DIC/SPCS high, medium and low dose groups (coccidiosis infection and treatment with different doses of micellar preparations), together with 10 healthy chickens used as uninfected control. The diet and drinking equipment of each experimental group were separately treated to prevent cross-infection. The same size trays were used to capture fecal material to assess drug treatment efficacy.

After 48 h of infection with coccidiosis, the infected groups were given drug treatment once a day for a total of 3 days. SPCS/DIC micelles were given high, medium and low doses of DIC. DIC suspension and PCS/DIC micelles were given at medium dosage, and uninfected groups were treated with the same amount of distilled water. The body weight gain (BWG), bloody diarrhea, intestinal lesion score and oocyst counts of ​​chickens infected with coccidiosis were investigated to evaluate the effect of treatment with different preparations. The average body weight of each group was recorded daily during the experiment, and the average weight change curve of each group of chicks was plotted. The body weight gain (BWG) was obtained by subtracting the initial average body weight from the final average body weight. The relative weight gain (RWG) of the uninfected control group was recorded as 100%, and that of the other groups was calculated according to the following Eq. (4). Based on the feed consumption and weight changes during the experiment, the feed conversion rate (FCR) of each group was calculated by Eq. (5):

4 $${\text{RWG(\% )}} = \frac{{{\text{Weight}}\;{\text{gain}}\;{\text{of}}\;{\text{experimental}}\;{\text{group}}}}{{{\text{Weight}}\;{\text{gain}}\;{\text{of}}\;{\text{uninfected}}\;{\text{group}}}} \times 100{\text{\% }}$$

5 $${\text{FCR(\% )}} = \frac{{{\text{Feed}}\;{\text{conversion}}}}{{{\text{Weight}}\;{\text{gain }}}} \times 100{\text{\% }}$$

Bloody diarrhea reflecting the severity of coccidiosis was monitored from day 5 to day 7 post-challenge. The merozoites of *E. tenella* reproduce in epithelial cells and would be released to intestinal tract once proliferation was completed. This causes bloody diarrhea due to rupture of the cecal cells, and bloody diarrhea is usually most severe after infection for 5 to 6 days. The intestinal lesion scores for each group were calculated, and different scores were assessed according to epithelial color, fluid accumulation and overall bowel appearance (0 = no lesions, 1 = mild lesions: few needle hemorrhages, normal thickness of the intestinal wall, 2 = moderate lesions: small amount of blood, slight increase in the thickness of the intestinal wall, some hemorrhages sites, 3 = severe lesions: swollen intestinal wall, large amount of blood, morphological cecum changes, 4 = more severe lesions: cecum atrophy, severe hypertrophy of the intestinal wall, blood clot). Fecal oocysts number was recorded on days 5, 6, and 7 after the onset of challenge by McMaster method. The average number of oocysts in 3 days is expressed as gram per oocyst production (OPG). At the autopsy of the chicken, cecal tissue samples were collected and fixed in 10% buffer paraformaldehyde. Tissue sections were stained, and the lesions such as hemorrhage, coccidial oocyst infection and villi erosion were observed under a microscope. Based on the relative weight gain rate, survival rate (SR), lesion value (LV) and oocysts value (OV), anti-coccidial index (ACI) was calculated according to Eq. (6).

6 $${\text{ACI}} = ( {\text{RWG}} + {\text{SR)}} - ( {\text{LV}} + {\text{OV)}}$$

### Statistical Analysis

All data obtained were presented as mean ± S.D. Significant differences were analyzed by Student’s *t* test using SPSS 23.0 statistical software (SPSS Inc., USA), where differences were considered significant (*p < 0.05, **p < 0.01, ***p < 0.001 and ****p < 0.0001).

## Results

### Synthesis and Structural Characterization of Conjugates

**Figure 1:**
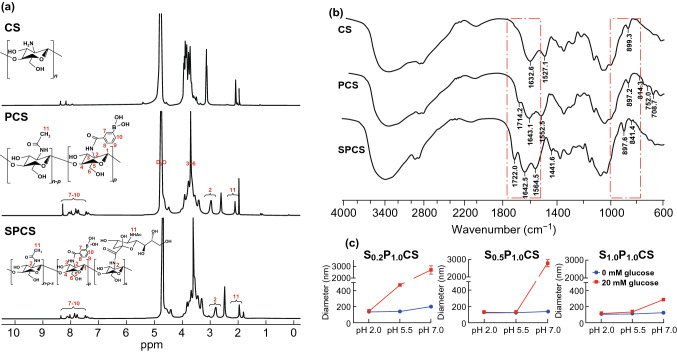
Structural characterization of various chitosan derivatives. ^1^H NMR spectra **a** and FTIR spectra **b** of CS, PCS and SPCS. **c** Diameter variations of SPCS micelles at pH values of 2.0, 5.5 and 7.0, with the presence and absence of glucose (20 mM)

To prepare the phenylboronic acid-rich and dual-responsive SA-PBA-CS conjugates (SPCS), EDC/NHS was used to combine CPBA and SA with CS polymer skeleton through amide reaction (Scheme 1). Firstly, CS polymer was modified with CPBA via amidation reaction to produce amphipathic PCS conjugates. Then, PCS copolymer was linked with SA moieties to endow SPCS conjugates with dual-sensitivity and targeting ability. The chemical structures were characterized and confirmed by ^1^H NMR and FTIR. As shown in Fig. 1a, the successful introduction of PBA moiety was confirmed by the peaks of benzene which appeared at 7.4–8.2 ppm. For SPCS conjugates, the characteristic peaks of SA protons appeared at 1.72 ppm, attributed to the methylene group of SA. Moreover, FTIR spectra of CS, PCS and SPCS are shown in Fig. 1b. Compared with CS copolymer which possessed an obvious peak at 899 cm^−1^ from β-(1,4) glucosidic bond and characteristic absorption peaks of CS at 1000–1150 cm^−1^, PCS conjugates displayed additional absorption peaks at 1720 cm^−1^ corresponding to stretching vibration of amide (C=O), 810 and 708 cm^−1^ corresponding to in-plane bending vibration of benzene (Ar–H) and 1442 cm^−1^ corresponding to stretching vibration of boric acid ester (B-O). The absorption band of SPCS conjugates at 2934 cm^−1^ was due to –CH_3_ of SA. The results confirmed the formation of amide bonds between CS and CPBA/SA. The polymer of PCSs was synthesized via changing the feeding amount of CPBA. The graft degree increased along with the feeding amount of CPBA moieties (Table S1) and reached its maximum when CPBA reacted with CS at a molar ratio of 1.5: 1. Polymers of P_1.0_CS and P_1.5_CS were applied for further conjugation with various amount of SA (Tables S2 and S3). The SPCSs could self-assemble micellar structure, of which the diameter changes were evaluated according to the variations of pH and glucose. Among SP_1.0_CS micelles, S_0.5_P_1.0_CS gave more desirable sensitivity profile of rapid swelling at pH 7.0 in 20 nM of glucose (Figs. 1c and S1), which was in accordance with cecum microenvironment, while the diameters of SP_1.5_CS micelles shared obvious increase at pH 5.5 (Figs. S2–S5), indicating that the particles might turn unstable in small intestine before reaching the lesions at cecum.

### Borate Bridge Formation and Critical Micelle Concentration of SPCS Conjugates

**Figure 2:**
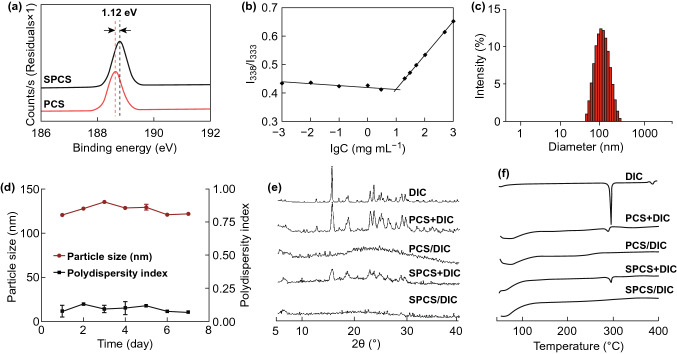
Physicochemical property and DIC-encapsulating capacity of SPCS micelles. **a** XPS spectra of PCS and SPCS. **b** Fluorescence intensity ratio (*I*_337_/*I*_332_) plot of pyrene versus logarithm of SPCS concentration (Log C) at pH 7.4. **c** The hydrodynamic particle size distribution of SPCS/DIC micelles measured by DLS. **d** Changes in diameter and PDI of SPCS/DIC micelles at 4 °C within a week. **e** XRD patterns, and **f** DSC curves of DIC, PCS + DIC physical mixture, PCS/DIC micelles, SPCS + DIC physical mixture and SPCS/DIC micelles

Borate bridge formation was essential for the construction of SPCS micelles. The X-ray photoelectron spectra (XPS) of PCS conjugates shared a peak at 188.64 eV, attributed to the free form of PBA (Fig. 2a), while the peak component of SPCS conjugates exhibited a higher binding energy by 1.12 eV compared with PCS, due to the chemical state variations of boron. Obviously, these results elucidated the formation of borates bridges in the micellar cores. To validate the capability of copolymers for compact and stable polymeric micelle formulation and the aggregation behavior in aqueous medium, critical micelle concentration (CMC) of amphiphilic PCS and SPCS conjugates was determined by fluorescence spectroscopy using pyrene as a probe. PCS copolymers exhibited a low CMC value of 17.78 μg mL^−1^, resulting from hydrophobicity induced by CPBA. As shown in Fig. 2b, CMC value of SPCS conjugates decreased to 10.58 μg mL^−1^ compared to that of PCS copolymers. The results indicated that the formation of reversible borate complexes by PBA and SA moieties could facilitate and stabilize micellization. Such low CMC value guarantees the self-assembled micelles to retain their original morphology under highly diluted conditions before reaching the targeting site, which is a significant advantage over low molecular weight surfactants. Based on the results above, SPCS conjugates possess great potential to be drug carrier for insoluble drugs as they can self-assemble stable micelles with hydrophobic inner cores.

### Preparation and Characterization of DIC-loaded Micelles

The SPCS micelles were prepared by a simple sonication method in aqueous solution followed by dialysis for 48 h to remove DMF. The non-functional PCS micelles were prepared by the same procedure as control. The micelles are characterized by primary physicochemical parameters including particle size, polydispersity index (PDI), drug loading (DL %) and encapsulation efficiency (EE %) (Table S4). Dynamic light scattering (DLS) measurements demonstrated that the diameter of SPCS/DIC micelles shrank from 139.5 nm to 124.1 nm (Fig. 2c) compared to PCS micelles. In accordance with diameter, PDI of DIC-loaded SPCS decreased from 0.153 to 0.092. Moreover, DL % and EE % of SPCS/DIC micelles were much higher than those of PCS/DIC micelles. These results above suggested more compact and stable hydrophobic inner core due to the borate formation by polyols of SA and PBA. The variations in diameter and PDI were further monitored (Fig. 2d). SPCS/DIC micelles exhibited negligible change in particle size and PDI within one week, suggesting the stabilization endows by borate cross-linkers.

To confirm the existing state of DIC in SPCS micelles, X-ray diffraction (XRD) and differential scanning calorimetry (DSC) are informative analysis for revealing physical properties of small molecules and polymer blends. The XRD pattern of DIC (Fig. 2e) demonstrated several diffraction peaks at 2θ of 15.5°, 18.6°, 22.8°, 23.5°, 28.8° and 29.5°, confirming its crystalline nature. For the physical mixture of PCS + DIC and SPCS + DIC, the DIC diffraction peaks remained to be observed. However, these DIC-specific peaks vanished in lyophilized PCS/DIC and SPCS/DIC micelles. The drug entrapment within the micelles can be proposed based on the complete absence of DIC diffraction peaks. The results could be attributed to the intense hydrophobic interactions between PBA moieties and DIC. Figure 2f illustrates DSC thermograms of free DIC, physical mixture of DIC + PCS and DIC + SPCS and medicated micelles. The thermogram of DIC revealed a sharp endothermic peak at 296 °C, indicating the melting of crystalline DIC. In the case of physical mixture of DIC + copolymers, the characteristic endothermic peak of DIC could still be observed; while the DIC peak was absent in the thermogram of drug-loaded micelles, confirming the DIC encapsulation into the micelles.

### In vitro Glucose and pH-triggered Micelles Disassembly

**Figure 3:**
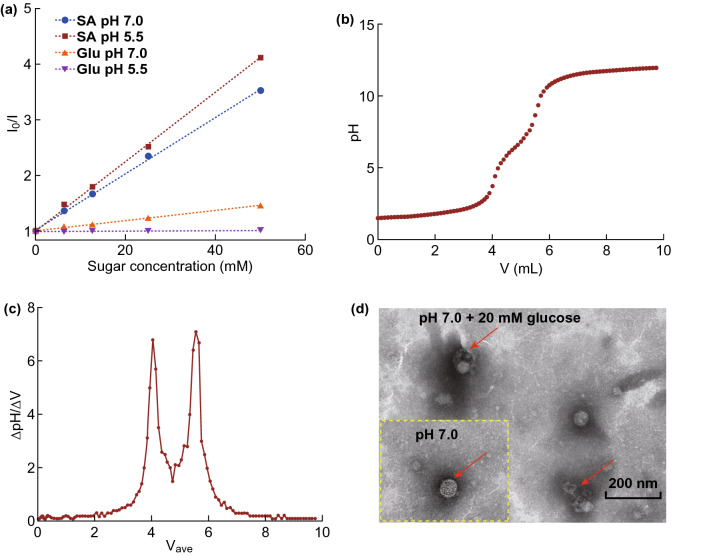
Stimuli-triggered physicochemical property transition. **a** Relative fluorescence as a function of sugar concentration for SPCS measured in phosphate buffer (0.1 M) at room temperature (*λ*_ex_ = 371 nm and *λ*_em_ = 425 nm) at pH 7.0 and 5.5. *I*_0_ and *I* represent the fluorescence intensity in absence and presence of sugar. **b** Titration curve against pH was measured at room temperature. **c** pKa of SPCS calculated by stair differential coefficient. **d** TEM images of SPCS/DIC micelles with and without the presence of glucose

To assess the reversible formation of borate bridge, we evaluated the binding affinity of PCS to molecules of SA and glucose by steady-state fluorescence measurement. In opposite to the earlier studies [17, 24] that shared fluorescence quenching along with the sugar addition, the fluorescence intensity of PCS conjugates increased in sync with sugar concentration. Accordingly, the electron-withdrawing carboxyl group would cause fluorescence quenching when connected to the benzene ring of phenylboronic acid, while the complex of sugar with PBA could weaken the quenching of carboxyl by conjugation effects. The data were fit by Stern–Volmer equation. Differently, we applied *I*/*I*_0_ instead of *I*_0_/*I*, demonstrating good linear relations as shown in Fig. 3a. Considering that the binding affinity of PBA to sugars is affected by pH, the experiments were performed at different conditions of pH 7.0 and 5.5. According to the regression equation, the conclusions could be achieved as follows. To displace SA moieties, the glucose concentration should exceed 763 mM at pH 5.5, which is impossible to reach in gastroenteric environments, suggesting the superior biostability of borate bridges. Oppositely, when pH approached 7, SA molecules could be substituted at a glucose concentration over 14 mM that could be easily realized at cecum site. Differentiated from the tetrahedral anionic borate bridges with carbohydrates like glucose, fructose or saccharose, a surprising stability in trigonal conformation was brought from multiple metastable binding sites along with intramolecular stabilization via B-O interactions between SA and PBA moieties [17]. To reveal the conformational switching of PBA moieties, we investigated the pKa of SPCS polymer by potentiometric titration. As shown in Fig. 3b, c, the pKa of SPCS conjugates was determined to be 6.84, indicating that a higher pH could trigger sugars to bind with PBA moieties on SPCS conjugates. The results above suggested that the borate bridge can maintain stable in acidic environment even with the presence of glucose, while these cross-linkers would be clipped by sugars like glucose at pH 7. In addition, the representative transmission electron microscopy (TEM) images of SPCS/DIC micelles demonstrated that the nanostructure was near-spherical in shape and well-distributed (Fig. 3d). The SPCS micellar size observed by TEM was ~ 90 nm which was a little smaller than that determined by DLS. These results could be ascribed to micellar shrink after water evaporation. To observe the glucose-triggered disintegration of the micellar structure, we dispersed the micelles in 20 mM glucose solution (pH 7.0) followed by TEM analysis. As indicated by the red arrow, the diameter of the micelles increased and the spherical structure became cracked and fragmented, indicating that the compact SPCS/DIC micelles collapsed because of glucose existence.

### In vitro Stimuli-triggered Drug Release and Micellar Stability against Surfactants

**Figure 4:**
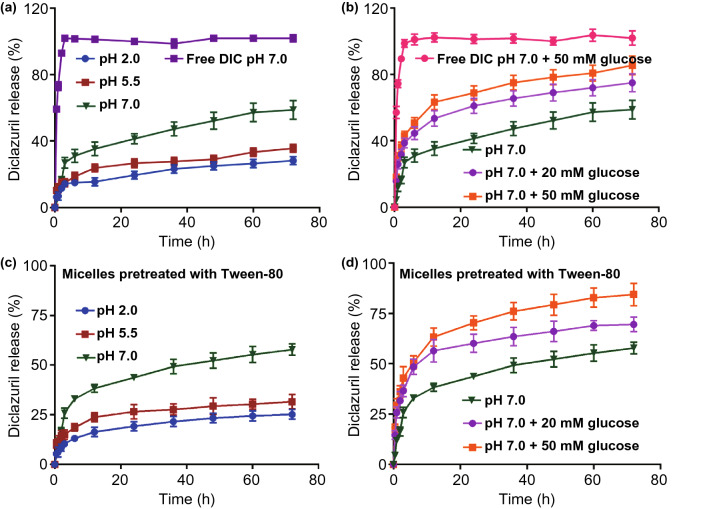
Stimuli-triggered drug release. **a** DIC release from SPCS/DIC micelles at different pH values of pH 2.0, pH 5.5 and pH 7.0. Free DIC release from dialysis bag at pH 7.0 was used as control. **b** DIC release profile from SPCS/DIC micelles in the presence of glucose at pH 7.0. Free DIC release from dialysis bag at pH 7.0 in the presence of 50 mM glucose was used as control. **c** SPCS/DIC micelle was preincubated with Tween 80 solution for 6 h and the DIC release profile at different pH values of pH 2.0, pH 5.5 and pH 7.0 was studied. **d** DIC release profile of the pretreated SPCS/DIC micelle in the presence of glucose at pH 7.0 was further studied. Data are expressed as mean ± SD, *n* = 3

To confirm intestinal stimuli-triggered drug release pattern upon high pH and concentrated glucose, the cumulative DIC release was investigated by dialysis method against different buffering media (Figs. 4a, b and S6). The inefficient and slow release of DIC was observed in acidic medium of pH 2 and 5.5, and the cumulative release was less than 30% within 72 h, nearly half of that at pH 7.0 (55.7%). The results were in accordance with Tween 80 preincubated SPCS/DIC micelles (Fig. 4c, d), implying hardly structure destruction under emulsified dissolution and intestinal media. The preferable drug release pattern was attributed to free amino group of chitosan in micelles, which was protonated at low pH for inhibition of micelle swelling and drug release, while amino deprotonation would alter ionic interaction among polymer chains at high pH. Moreover, the release speed of DIC was dramatically accelerated in the presence of glucose (20 or 50 mM) for mimicking gut environment, and the cumulative DIC release reached 67% and 85%, respectively. Structurally, PBA moieties could react with polyhydroxy groups of glucose and form soluble phenyl borates, which destroyed the hydrophobic core of SPCS/DIC micelles and promoted the drug release. As reported, the extremely insoluble drugs were difficult to release from the micellar core, for instance, 90% of the total during 10 day-release period [25]. For coccidiosis treatment, the sustained release profile is not favorable due to the susceptibility of drug resistance. However, the therapeutic concentration could be achieved rapidly by burst release of DIC molecules for enhanced insecticidal efficacy and reduced side effects.

**Figure 5:**
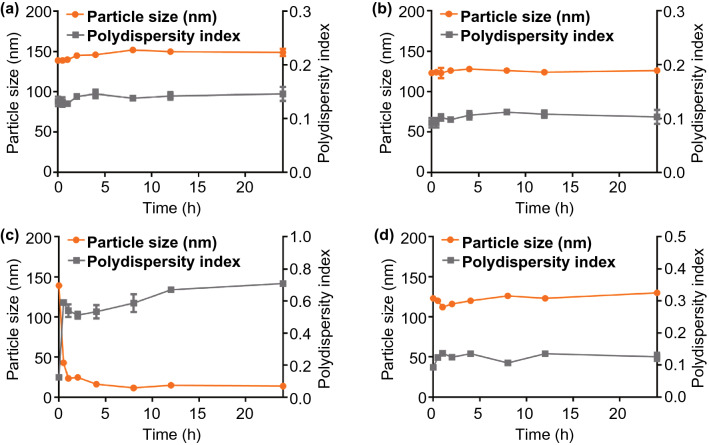
Stability of micellar structure against surfactants. Diameter and PDI variations of **a** PCS/DIC micelles and **b** SPCS/DIC micelles incubated with 0.5% Tween 80 solution. Diameter and PDI variations of **c** PCS/DIC micelles and **d** SPCS/DIC micelles incubated with 2% Tween 80 solution. Data are expressed as mean ± SD, *n* = 3

Moreover, the stability of micellar structure against surfactants was further investigated by monitoring the variation of particle size. Both of PCS/DIC and SPCS/DIC micelles were stable without DIC liberation at 0.5% of Tween 80 concentration within 24 h (Fig. 5a, b). When surfactant concentration increased to 2%, the particle size of PCS/DIC decreased sharply to ~ 20 nm and the polydispersity index increased over 0.7, while that of SPCS micelles remained stable (Fig. 5c, d). The intact structure could be ascribed to the borate cross-linkages in micellar core. These results suggested that SPCS/DIC micelles were likely to be integrated in the upper gastrointestinal tract without drug leakage after oral administration, whereas rapid drug release could be achieved at cecum site that was infected severely by coccidium. Therefore, SPCS micelles are highly promising drug delivery system to achieve site-specific rapid release of anti-coccidial drugs.

### In vitro Safety Profile and Cellular Internalization

**Figure 6:**
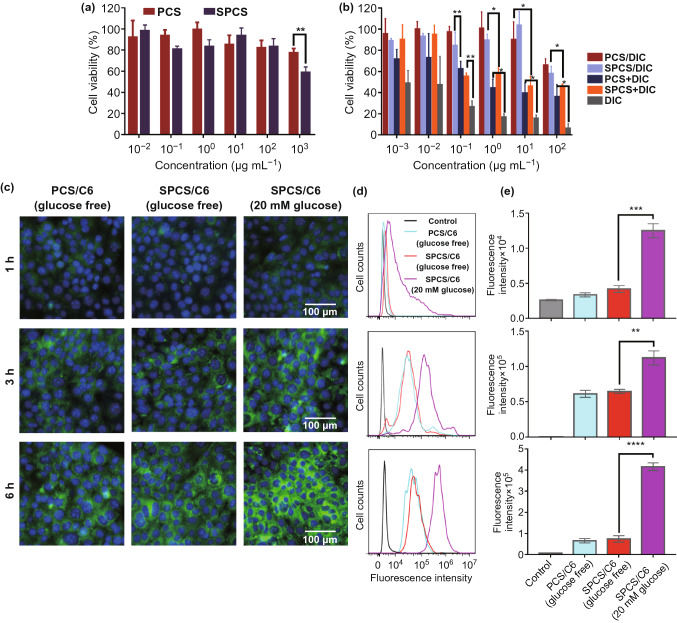
In vitro safety profile and cellular internalization. **a** Cytotoxicity of chitosan derivative and **b** DIC-encapsulated micelles against Caco_2_ cells with different concentrations for 48 h. **c** Inverted fluorescence microscope images of PCS/C6 and SPCS/C6 micelles in Caco_2_ cells under glucose free condition, and Caco_2_ cells inculcated with SPCS/C6 at a glucose concentration of 20 mM. **d** Representative FACS images and **e** quantitative analysis of intracellular internalization of PCS/C6 and SPCS/C6 in Caco_2_ cells at 1, 3 and 6 h. Data are expressed as mean ± SD, *n* = 3. *p < 0.05, **p < 0.01, ***p < 0.001, ****p < 0.0001

The cytotoxicity of materials and nanomedicine is a very important property that restricts practical application. Therefore, the in vitro cytotoxicity of blank micelles (PCS or SPCS) and DIC-encapsulated micelles (PCS/DIC, SPCS/DIC) against Caco_2_ cells was estimated by MTT assay. As demonstrated in Fig. 6a, cell viability of various blank micelles was approximately 100% even at a high concentration of 100 μg mL^−1^. A slight decrease in cell viability was observed when the concentration reached 1000 μg mL^−1^. The results suggested that PBA-installed chitosan and the corresponding blank micelles shared negligible cytotoxicity to Caco_2_ cells and potential application for drug delivery. Free DIC revealed a dose-dependent cytotoxicity against Caco_2_ cells (Fig. 6b) and less than 10% cells survived at a high DIC concentration of 100 μg mL^−1^, while the physical mixture of drug and carrier shared lower cytotoxicity than DIC. The results might generate from micellar self-assembly in aqueous medium. The drug-contained micelles displayed significantly lower cytotoxicity compared with that of free DIC and physical mixture groups, which was possibly ascribed to the core–shell structure of the drug-loaded micelles. These findings of cytotoxicity assay indicated that SPCS/DIC micelles can notably reduce toxicity of free DIC and avoid intestinal cell damages.

During coccidium infection, cytoclasis and metabolic waste of coccidium would cause severe inflammation at the infected intestinal segment. The stimulated gastrointestinal cells would upregulate E-selectin expression, a type of adhesion protein to facilitate leukocytes emigration from the bloodstream. Accordingly, SA molecules share a high affinity to E-selectin and could be introduced as targeting ligand for the infected cells. The pH and glucose-activated cellular internalization of SPCS/C6 micelles was examined by inverted fluorescence microscope in a colon cancer Caco_2_ cell line with E-selectin expressing on the cell membrane. As expected, under normal cell culture condition of 25 mM glucose, SPCS/C6 micelles demonstrated much stronger fluorescence in Caco_2_ cells compared to PCS/C6 micelles (Fig. 6c). However, in sugar-free culture medium, the fluorescence of SPCS/C6 decreased that was similar to PCS/DIC group. These results suggested that glucose could displace SA molecules and transfer SA onto the surface of SPCS micelles, followed by E-selectin-mediated conglutination and internalization by Caco_2_ cells. Simultaneously, the drug release could be accelerated due to the stable combination of PBA with glucose in the core. Furthermore, the fluorescence intensity of C6 gradually increased during 6 h incubation, indicating that the cellular uptake of the micelles increased in a time-dependent manner. The intracellular internalization of SPCS/C6 micelles was further quantitatively investigated by flow cytometry (FCM). After 6 h incubation, the mean fluorescence intensity of SPCS/C6 micelles was approximately sixfold higher than that of PCS/C6 group with the presence of glucose (Fig. 6d, e), while no significant difference was observed without glucose in the culture medium. The results also confirmed the pH and glucose-triggered SA exposure and enhanced cellular internalization for further anti-coccidiosis therapy.

### Pharmacokinetics Studies

**Figure 7:**
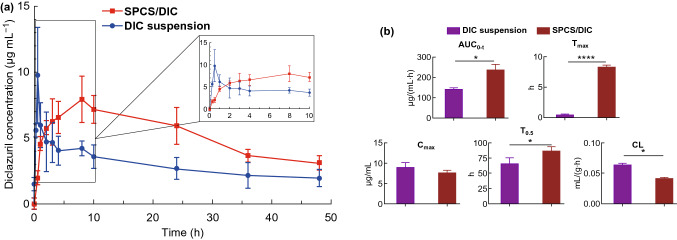
**a** Mean DIC plasma concentration (μg mL^−1^) following a single dose oral administration (15 mg kg^−1^) of SPCS/DIC micelle and DIC suspension. **b** Pharmacokinetics parameters following a single oral administration (15 mg kg^−1^ of DIC) of DIC suspension and SPCS/DIC micelles. Data represent the mean ± SD, *n* = 6. *p < 0.05 and ****p < 0.0001 as compared to DIC suspension

For pharmacokinetic studies, the plasma drug concentration–time curves demonstrated that DIC in SPCS micelles and CMC-Na suspensions revealed a similar metabolism process in vivo following oral administration at a single dose of 15 mg kg^−1^ (Fig. 7a). The pharmacokinetic process could be divided into the absorption and distribution stages. When the drug directly ingested into the gastrointestinal tract, the rate of drug absorption into the blood circulation was higher than that of the distribution process, and then, the drug concentration gradually increased to reach the maximum. Meanwhile, the drug absorption rate was less than that of the distribution, and SPCS/DIC micelles could maintain a higher plasma concentration than that of DIC suspension, suggesting a prolonged retention of the sufficient concentration of drugs. As shown in Fig. 7b and Table S5, *T*_max_ and AUC_0−t_ values of SPCS/DIC micelles were 8.25 h and 236.10 µg mL^−1^ h^−1^, significantly higher than that of drug suspension by approximately 17- and 1.7-folds. There was no statistically significant difference in the half-life and *C*_max_ values of the two preparations. The results demonstrated that SPCS/DIC micelles could be applied as a long-acting formulation to reduce the frequency of drug administration and the toxic side effects due to the peak-valley phenomenon of plasma drug concentration.

### In Vivo Biodistribution and anti-coccidial Efficacy

**Figure 8:**
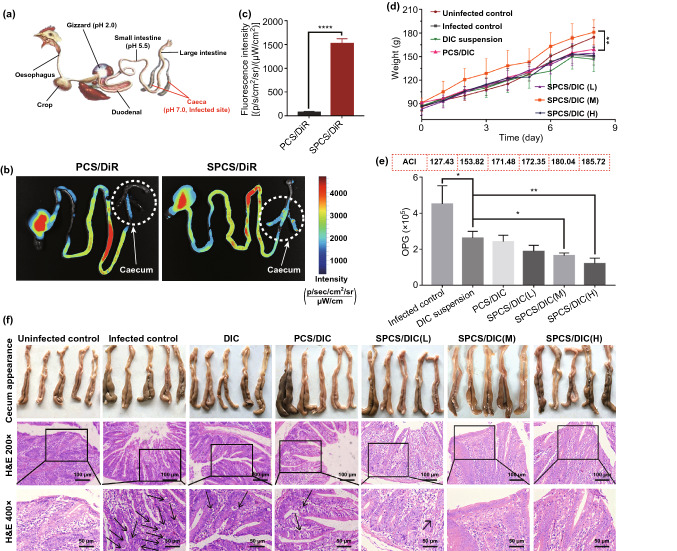
In vivo biodistribution and anti-coccidial efficacy. **a** Schematic diagram of chicken digestive system and corresponding microenvironments at different sections. **b** Representative ex vivo images of intestinal tract at 4 h after oral administration of PCS/DiR and SPCS/DiR micelles, respectively. **c** Quantification of fluorescence intensity in cecum. **d** Average weight change of the chicks administrated by DIC suspension, PCS/DIC and SPCS/DIC (L, M, H) micelles, with the infected and uninfected groups as controls. **e** Number of oocysts per gram of feces (OPG) and ACI values representing anti-coccidial capacity of different DIC formulations. **f** Cecum appearance and H&E staining images were collected after the experiments. Data are expressed as mean ± SD, *n* = 10. *p < 0.05, **p < 0.01 and ****p < 0.0001

Once infected with coccidia, the contraction amplitude of cecum would increase, restraining the internalization of colonic contents by cecum. Moreover, exudates form the damaged intestinal wall that would deposit in cecum and even form tuberculosis of cecum, which may block the cecal entrance. These pathological changes could prevent the drugs from reaching the cecum, leading to difficulty for coccidia treatment. The previously studied and commercially available coccidiostat preparations have revealed insufficient lesion targeting due to the rapid drug elimination. Hence, bioadhesive CS polymer and SA molecules were applied in SPCS/DIC preparations for prolonged digestive system retention and increased lesion-specific accumulation via superior affinity between SA and E-selectin overexpressed on infected cells. To evaluate the cecum-targeting efficiency of SPCS micelles, the near-infrared dye DiR encapsulated micelles including SPCS/DiR and PCS/DiR were orally administrated to the coccidiosis-infected chickens. The intestinal tracts were harvested, and the fluorescence distribution in the tracts was monitored at 4-h post-administration (Fig. 8a, b). The SPCS/DiR group shared an obvious fluorescence signal at the cecum site, while group of PCS/DiR gave a negligible signal. According to quantitative region-of-interest analysis (Fig. 8c), the fluorescence intensity of cecum from SPCS/DiR group was about 20-fold higher than that of PCS/DiR. These results indicated that SA installation endowed SPCS micelles with cecum targetability by position transition and E-selectin recognition.

The anti-coccidial parameters for different groups are shown in Table S6, including weight gain, relative body weight gain and feed conversion rate (FCR). Among the parameters, weight gain and FCR represent the animal growth most intuitively, and FCR values can also be introduced to evaluate the health conditions of digestive systems. Generally, low FCR values indicated that the animals were well nourished accompanied with good function of main organs. From Table S6, the uninfected chickens gained weight by 81% in 8 days with a low FCR of 3.06. Oppositely, the weight gain rate of the positive control group was only 43% and FCR value increased to 5.42, suggesting serious damages to chicken’s health. The weight variations were further monitored during the therapeutic process (Fig. 8d). For the treated groups, the order of curative effects was as follows, DIC suspension < SPCS/DIC (L) < PCS/DIC < SPCS/DIC (H) < SPCS/DIC (M) according to the weight gain and FCR values. Compared with commercial DIC suspension, the weight gain of SPCS/DIC (M) group increased by 56.5% and the corresponding FCR decreased from 4.92 to 3.66. Surprisingly, the middle DIC dose in SPCS micelles gave the highest curative effects, indicating that a high DIC intake would delay the chicken organ developments. Moreover, the non-bridged PCS/DIC micelles shared a lower weight gain than SPCS/DIC group, demonstrating that SA moieties could facilitate the cecum retention to enlarge the curative efficiency. The enhanced anti-coccidial efficacy from SPCS/DIC could be attributed to the accelerated dissolution by the dual-stimuli responsive drug-micelles.

The bloody diarrhea numbers of different groups during 5–7 days after infection are demonstrated in Table S7. Obviously, the order of total bloody feces was infected control > DIC suspension > SPCS/DIC (L) > PCS/DIC > SPCS/DIC (M) ≈ SPCS/DIC (H). The results indicated that SPCS/DIC micelles could alleviate the intestinal injuries efficiently and the medium dose was enough to reach desirable response. We further examined the lesions of intestinal tracts (Table S8), and the lesion scores revealed a similar order with the bloody feces, confirming the enhanced anti-infection of rationally designed SPCS/DIC micelles. As shown in Fig. 8e, the order of oocysts number per gram of feces (OPG) was demonstrated in accordance with that of bloody feces. The middle and high-dose groups induced significant decrease in OPG values compared with commercial DIC suspension. The anti-coccidial index (ACI) of each group was calculated based on the data mentioned above (Fig. 8e). Groups of SPCS/DIC (M) and SPCS/DIC (H) gave the highest ACI values of 180.04 and 185.72 that belonged to highly effective coccidiostats according to the grade estimation (Table S9). The ACI value of PCS/DIC micelles was close to that of SPCS/DIC (L) group within the range of moderately effective coccidiostats, while DIC suspension gave an ACI value below 160, much lower than that of micellar preparations. The images of cecum and the corresponding H&E staining are demonstrated in Fig. 8f. The ceca of uninfected group were in normal size and white smooth appearance, and the corresponding H&E staining performed no mucosal lesion and coccidial oocyst. In the infected group, the ceca revealed a swollen dark appearance with numerous irregular mottled hemorrhage areas on the serosal surface. Moreover, incrassation of intestinal walls, bowel injuries and numerous round coccidial oocysts (as black arrows indicated) could be observed in the photographs of H&E staining. In the treated groups, the pathologic changes were alleviated to various degrees, similar to the order of OPG values. No oocysts were found on the H&E staining sections of SPCS/DIC (M) and SPCS/DIC (H) treated groups. Collectively, the results suggested that SPCS/DIC micelles could eliminate the coccidia in cecum effectively and promote the recovery of intestinal injury at a relatively low dosage, where the introduction of borate bridges was the key for amplifying anti-coccidial efficacy.

## Discussion

Triazine benzacetonitril drugs hold great potential in coccidiosis infection treatment with broad spectrum and high efficacy. However, their clinical use in livestock has historically been hampered by poor solubility and drug ability. Herein, we have demonstrated the rational design of a combinative assembly strategy inspired reversibly borate-bridged polymeric micelles. The micelles were fabricated with PBA and SA dual-modified SPCS conjugates (Fig. 1) to serve as a promising nanocarrier for local infection treatment. Due to the coaction of hydrophobicity driving and borate bridging, SPCS polymers could spontaneously fabricate micellar structure together with efficient DIC encapsulation (Fig. 2). The specific borate bridges generated from SA and PBA moieties remained stable even at pHs below the pKa of PBA molecules, which protected the configuration from disassembly in acidic upper gastrointestine (Fig. 3). As previously reported, the electron-donating group could make a coordination bond (B ← :N) with the boron atom to stabilize the complex of PBA with polyol. Thus, SA installed amino-rich CS copolymer could give an enhanced affinity to PBA. When triggered with sugars in digestive tract, the corresponding borates would become unstable and sensitive to hydrolysis along with the pH decrease. The SPCS micelles performed a dual-stimuli responsive drug release profile (Fig. 4), and the affinity between sugars and PBA moieties could soar at a higher pH, leading to borate bridge rupture by competitive binding.

*Eimeria tenella* infection could cause severe damages to cecum, accompanied by a series of symptoms such as bloody diarrhea, anemia, anorexia and growth retardation. Once infected, the coccidia would spread in the intestinal tract and inside the intestinal epithelial cells. On market, the preparations are merely focused on increasing the drug solubility; however, the specific lesion accumulation and retention and the high drug intake within infected cells are of great importance for the efficient anti-coccidial therapy. In this study, SPCS/DIC micelles were designed for filling the vacancy. When reaching the cecum, SA groups in SPCS structure could expose to the micellar surface, which endowed the terminal micelles with prolonged drug retention and enhanced cellular internalization to eliminate the pathogen inside cells (Fig. 6). Moreover, a high affinity to the infected cells for SA molecules could be ascribed to the elevated E-selectin expression, a type of adhesion protein from severe inflammation induced by cytoclasis and coccidium metabolite.

In breeding industry, the applicable preparations of anti-coccidial drugs are usually added into potable water or fodder, and the animals could be free to take in drug at random. As a result, the preparations should be of high safety within a wide range of doses. SPCS copolymer and the DIC-encapsulated micelles elicited negligible cytotoxicity even at a high dose, for potential large-scale applications in livestock and poultry industry (Fig. 6). Moreover, the bioavailability of DIC was notably improved by the micellar formulation (Fig. 7). After treatment, SPCS/DIC micelles significantly relieved the coccidiosis infection and its complications, generating rapid weight gain and secondary infection prevention (Fig. 8). This is in stark contrast to the non-bridged PCS/DIC micelles and DIC suspension that yielded poor response in the infected chicken. Due to the structural disassembly and payload leakage before reaching the lesions, the unblocked PBA groups in PCS/DIC micelles might be easier to bind with the sugars, resulting in the disturbance of hydrophobic micellar core and drug stable shielding. Consequently, the borate-bridged SPCS micelles could give an evident enhancement in bioavailability and bioeffectiveness.

## Conclusion

In this study, we have demonstrated the rational design of a combinative assembly strategy inspired reversibly borate-bridged polymeric micelles. The borate-bridged SPCS/DIC micelles demonstrated remarkable biostability, biosafety and flexible drug release profile for local infection treatment and provided an enhanced anti-coccidial efficacy in vivo. Our findings suggested that the combinative assembly polymeric micelles paved a new path for prevention and treatment of coccidiosis.

## Electronic supplementary material

Below is the link to the electronic supplementary material.

Supplementary material 1 (PDF 349 kb)
