# Supplementary material for: A Combinative Assembly Strategy Inspired Reversibly Borate-Bridged Polymeric Micelles for Lesion-Specific Rapid Release of Anti-Coccidial Drugs
Source: Nanomicro Lett. 2020 Jul 25;12:155. doi: 10.1007/s40820-020-00495-1 (PMC7770674; doi:10.1007/s40820-020-00495-1)
Supplement: Supplementary file 1 — Supplementary material 1 (PDF 349 kb) [file 40820_2020_495_MOESM1_ESM.pdf]

Supporting Information for

# A Combinative Assembly Strategy Inspired Reversibly Borate-Bridged Polymeric Micelles for Lesion-Specific Rapid Release of Anticoccidial Drugs

Hao Cheng[1,#], Huaqing Zhang[1,#], Gujun Xu[1], Jin Peng[1], Zhen Wang[1], Bo Sun[2], Djamila Aouameur[1], Zhechen Fan[1], Wenxin Jiang[1], Jianping Zhou[1,*], Yang Ding[1,*]

[1]State Key Laboratory of Natural Medicines, Department of Pharmaceutics, China Pharmaceutical University, 24 Tongjiaxiang, Nanjing 210009, People's Republic of China

[2]Department of Radiation Oncology, Lineberger Comprehensive Cancer Center, Carolina Center for Cancer Nanotechnology Excellence, Carolina Institute of Nanomedicine, University of North Carolina at Chapel Hill, Chapel Hill, NC 27599, USA

[#]Hao Cheng and Huaqing Zhang contributed equally to this work

*Corresponding authors. E-mail: zhoujianp60@163.com (J. Zhou); dydszyzf@163.com (Y. Ding)

## Supplementary Figures

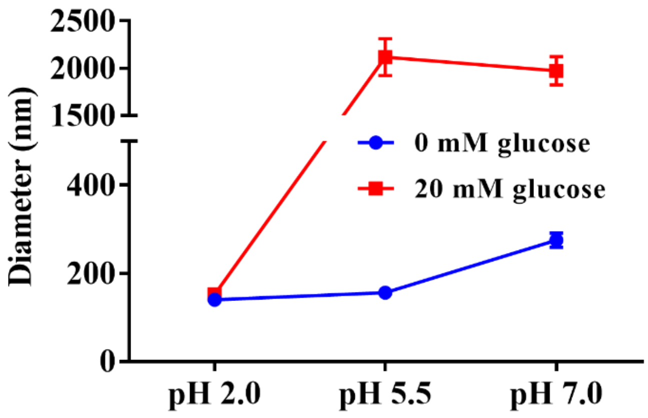


**Fig. S1** Diameter variations of $S_{0.1}P_{1.0}$CS/DIC micelles at pH values of 2.0, 5.5 and 7.0, with the presence and absence of glucose (20 mM). Data are expressed as mean±SD, n=3

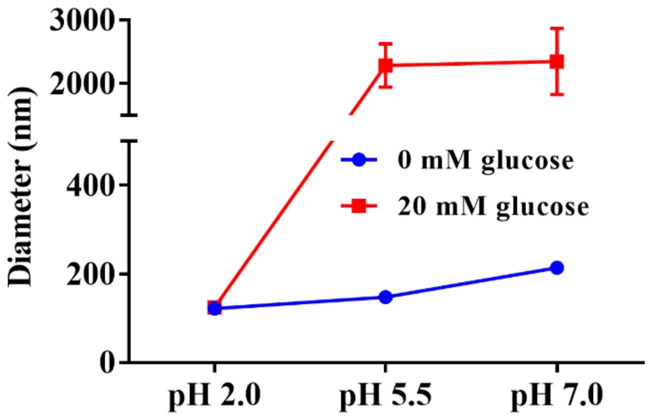


**Fig. S2** Diameter variations of $S_{0.1}P_{1.5}$CS/DIC micelles at pH values of 2.0, 5.5 and 7.0, with the presence and absence of glucose (20 mM). Data are expressed as mean±SD, n=3

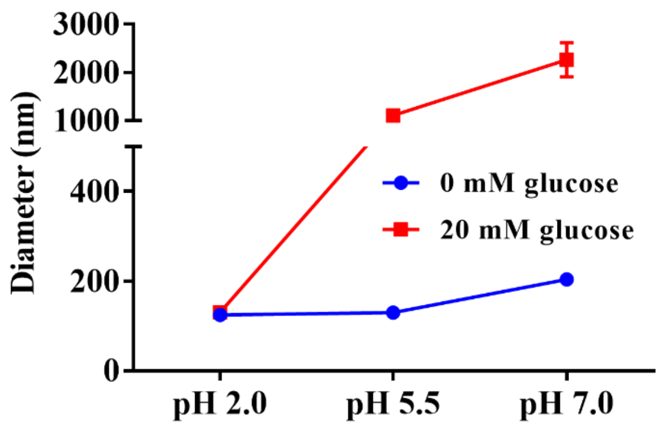


**Fig. S3** Diameter variations of $S_{0.2}P_{1.5}$CS/DIC micelles at pH values of 2.0, 5.5 and 7.0, with the presence and absence of glucose (20 mM). Data are expressed as mean±SD, n=3

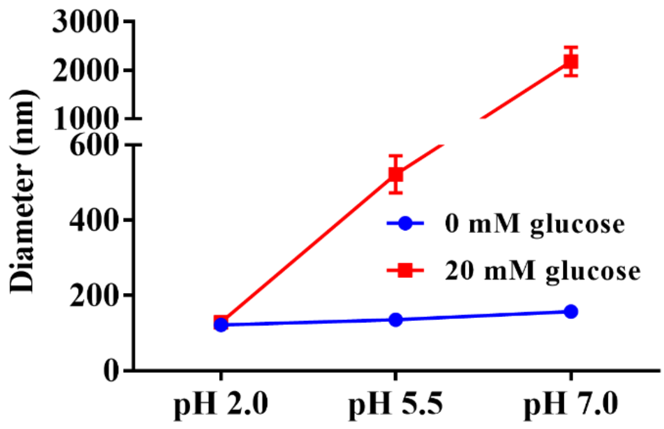


**Fig. S4** Diameter variations of $S_{0.5}P_{1.5}$CS/DIC micelles at pH values of 2.0, 5.5 and 7.0, with the presence and absence of glucose (20 mM). Data are expressed as mean±SD, n=3

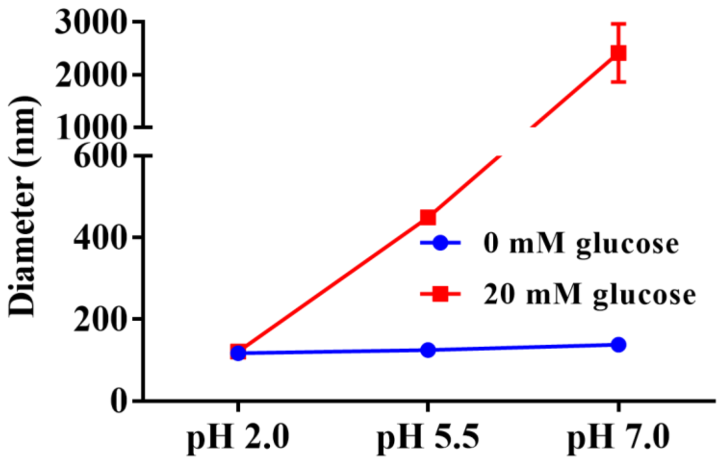


**Fig. S5** Diameter variations of $S_{1.0}P_{1.5}$CS/DIC micelles at pH values of 2.0, 5.5 and 7.0, with the presence and absence of glucose (20 mM). Data are expressed as mean±SD, n=3

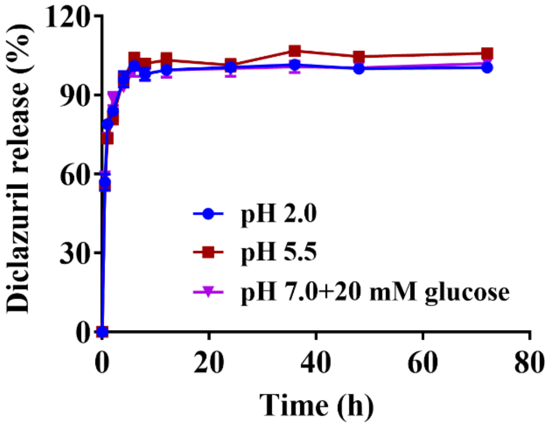


**Fig. S6** Free DIC release profile from the dialysis bag at different pH values of 2.0, 5.5, and in the presence of glucose (20 mM) at pH 7.0. Data are expressed as mean±SD, n=3

**Table S1** Graft ratio of PBA moieties in $P_xCS$ copolymers, where $x$ is the ratio of PBA molecules to CS monomers. Data are presented as mean ± SD, n = 3

| Type of $P_xCS$ | PBA: CS (n/n) | Graft ratio (%) |
|---|---|---|
| $P_{0.5}CS$ | 0.5 | 11.93 ± 1.56 |
| $P_{1.0}CS$ | 1.0 | 35.13 ± 3.89 |
| $P_{1.5}CS$ | 1.5 | 40.45 ± 2.14 |
| $P_{2.0}CS$ | 2.0 | 41.38 ± 3.24 |

**Table S2** Graft ratio of SA moieties in $S_yP_{1.0}CS$ copolymers, where $y$ is the ratio of SA moieties to PBA molecules. Data are presented as mean ± SD, n = 3

| Type of $S_yP_{1.0}CS$ | SA: PBA (n/n) | Graft ratio (%) |
|---|---|---|
| $S_{0.1}P_{1.0}CS$ | 0.1 | 4.57 ± 1.16 |
| $S_{0.2}P_{1.0}CS$ | 0.2 | 14.56 ± 3.58 |
| $S_{0.5}P_{1.0}CS$ | 0.5 | 21.31 ± 2.79 |
| $S_{1.0}P_{1.0}CS$ | 1.0 | 25.05 ± 4.18 |

**Table S3** Graft ratio of SA moieties in $S_yP_{1.5}CS$ copolymers, where $y$ is the ratio of SA moieties to PBA molecules. Data are presented as mean ± SD, n = 3

| Type of $S_yP_{1.5}CS$ | SA: PBA (n/n) | Graft ratio (%) |
|---|---|---|
| $S_{0.1}P_{1.5}CS$ | 0.1 | 4.14 ± 0.93 |
| $S_{0.2}P_{1.5}CS$ | 0.2 | 10.68 ± 2.76 |
| $S_{0.5}P_{1.5}CS$ | 0.5 | 16.67 ± 2.63 |
| $S_{1.0}P_{1.5}CS$ | 1.0 | 17.01 ± 3.32 |

**Table S4** Characterization of series of DIC-loaded micelles. Data are presented as mean ± SD, n = 3. *p < 0.05 and ***p < 0.001

| Samples | Size (nm) | PDI | DL (%) | EE (%) |
|---|---|---|---|---|
| SPCS/DIC | 124.1 ± 0.9 | 0.092 ± 0.008 | 13.4 ± 1.16* | 74.3 ± 4.2*** |
| PCS/DIC | 139.5 ± 1.1 | 0.153 ± 0.013 | 8.97 ± 1.59 | 59.1 ± 4.3 |

**Table S5** Pharmacokinetics parameters following a single oral administration (15 mg/kg of DIC) of DIC suspension and SPCS/DIC micelles. Data are presented as mean ± SD, n = 6. *p < 0.05 and ****p < 0.0001 as compared to DIC suspension

| Parameters | Unit | DIC suspension | SPCS/DIC |
|---|---|---|---|
| $AUC_{0\text{-}t}$ | $\mu g{\cdot}mL^{-1}h$ | 140.40 ± 7.07 | 236.10 ± 28.69* |
| $C_{max}$ | $\mu g{\cdot}mL^{-1}$ | 8.90 ± 1.20 | 7.50± 0.63 |
| $T_{max}$ | h | 0.48 ± 0.03 | 8.25 ± 0.35**** |
| $t_{1/2}$ | h | 64.56 ± 10.55 | 85.85 ± 7.59* |
| CL | $mL{\cdot}(h{\cdot}g)^{-1}$ | 0.063 ± 0.0034 | 0.041 ± 0.0026* |

**Table S6** Body weight determined at the initial and the end of the experiment, and relative body weight gain (%), feed conversion ratio (FCR). Data are expressed as mean ± SD, n=10

| Groups | Body weight | | Weight gain (%) | Relative body weight gain (%) | Feed conversion ratio (FCR) |
|---|---|---|---|---|---|
| | Before challenge (g) | After challenge (g) | | | |
| Negative control | 95.8 ± 5.7 | 173.3 ± 13.8 | 81 | 100 | 3.06 |
| Positive control | 96.4 ± 4.8 | 138.2 ± 14.3 | 43 | 53 | 5.42 |
| SPCS/DIC (H) | 92.8 ± 7.6 | 154.5 ± 9.2 | 67 | 82 | 3.63 |
| SPCS/DIC (M) | 94.7 ± 5.9 | 163.0 ± 27.6 | 72 | 89 | 3.66 |
| SPCS/DIC (L) | 93.9 ± 7.0 | 151.4 ± 16.1 | 61 | 76 | 4.02 |
| PCS/DIC | 96.4 ± 7.2 | 153.9 ± 21.6 | 60 | 74 | 3.88 |
| DIC | 96.3 ± 5.8 | 141.0 ± 17.9 | 46 | 57 | 4.92 |

**Table S7** Bloody faeces counted during 5-7 days after challenge with Eimeria tenella. Data are expressed as pooled bloody faeces from 10 chicks in each group

| Groups | Days after challenge | | | Total bloody faeces |
|---|---|---|---|---|
| | 5 | 6 | 7 | |
| Negative control | 0 | 0 | 0 | 0 |
| Positive control | 11 | 6 | 6 | 23 |
| SPCS/DIC (H) | 6 | 3 | 3 | 12 |
| SPCS/DIC (M) | 5 | 5 | 3 | 13 |
| SPCS/DIC (L) | 4 | 6 | 7 | 17 |
| PCS/DIC | 4 | 6 | 6 | 16 |
| DIC | 6 | 8 | 5 | 19 |

**Table S8** Intestinal lesions examined at 8 days after challenge with Eimeria tenella. Data are expressed as mean ± SD, n=10

| Groups | Intestinal lesion ranks | | | | | Lesion scores (mean ± SD) |
|---|---|---|---|---|---|---|
| | 0 | 1 | 2 | 3 | 4 | |
| Negative control | 8 | 0 | 0 | 0 | 0 | 0.00±0.00 |
| Positive control | 5 | 1 | 2 | 0 | 0 | 0.62±0.92 |
| SPCS/DIC (H) | 7 | 1 | 0 | 0 | 0 | 0.12±0.35 |
| SPCS/DIC (M) | 7 | 1 | 0 | 0 | 0 | 0.12±0.46 |
| SPCS/DIC (L) | 6 | 1 | 1 | 0 | 0 | 0.38±0.46 |
| PCS/DIC | 5 | 3 | 0 | 0 | 0 | 0.37±0.35 |
| DIC | 5 | 0 | 2 | 0 | 0 | 0.51±0.71 |

**Table S9** Grade estimation of coccidiostats according to ACI values

| ACI | >180 | 160-180 | 120-160 | <120 |
|---|---|---|---|---|
| Grade of coccidiostats | Highly effective | Moderately effective | Inefficient | Invalid |
